# The influence of gender and body mass index on the FPI-6 evaluated foot posture of 10- to 14-year-old school children in São Paulo, Brazil: a cross-sectional study

**DOI:** 10.1186/s13047-016-0183-0

**Published:** 2017-06-27

**Authors:** Barbarah Kelly Gonçalves de Carvalho, Patrícia Jundi Penha, Nárima Lívia Jundi Penha, Rodrigo Mantelatto Andrade, Ana Paula Ribeiro, Sílvia Maria Amado João

**Affiliations:** 1 Faculty of Medicine, University of São Paulo, Department of Physical Therapy, Speech and Occupational Therapy, Musculoskeletal Evaluation Laboratory, University of São Paulo, Rua Cipotânea, 51, Cidade Universitária, CEP: 05360-160 São Paulo, SP Brazil; 2 Department of Physical Therapy, Faculty of Human Sciences and Health, Pontifícia Universidade Católica de São Paulo, São Paulo, Brazil; 3 Investigator in the Musculoskeletal Evaluation Laboratory, Department of Physical Therapy, Speech and Occupational Therapy, University of São Paulo, São Paulo, Brazil; 4 Department of Physical Therapy, Faculty of Medicine, University of Santo Amaro, São Paulo, Brazil

**Keywords:** Posture, Evaluation, Feet, Adolescent

## Abstract

**Background:**

Adolescence is marked by changes to the body, including the feet. The Foot Posture Index (FPI-6) stands out from other foot type classification methods as valid, reliable, and multidimensional. However, the current literature differs according to age group, with little consolidation of normative data in school children, largely due to the influence of such factors as sex, age and body mass index (BMI). Thus, this study assesses foot posture in adolescents according to age, sex and BMI.

**Methods:**

The study evaluated 1.394 adolescents from Amparo and Pedreira regions in São Paulo, Brazil. Subjects were positioned barefoot on a wooden base and each foot was assessed by FPI-6 criteria. Each criterion was scored on a scale of −2 to +2, negative for supinated and positive for pronated posture. Initially the data were assessed for normality using the Shapiro-Wilk test and descriptive statistics were calculated. To investigate and compare the scores of FPI-6 with regards to age and body mass index, analysis of variance (ANOVA) was used, followed by post hoc Tukey. To compare the FPI-6 with regard to gender, an independent student *t test* was used. All data were analyzed using SPSS version 21.0 and the 5% significance level.

**Results:**

Boys had higher scores than girls (*p* = 0.037) for the right foot, and the group with normal BMI values scored higher than the obese group (*p* = 0.001). For the left foot, 11- and 13-year-olds differed (*p* = 0.024) with respect to age in general. The overweight and obese group scored lower than the normal BMI group (*p* = 0.039; *p* = 0.001, respectively).

**Conclusions:**

Overall, the feet in this study were classified as normal, with a tendency to pronation, particularly in boys. There were differences between the 11 and 13 year groups and, with regard to BMI, there were higher scores for the group with normal BMI. Therefore, a higher BMI in adolescence is not indicative of a pronated foot type.

## Background

The arthrokinematic characteristics of foot misalignment compromises the foot’s static and dynamic nature and its capacity to sustain body weight and distribute plantar pressure [1]. The poor posture of the foot and its misalignment increases the risk of injury to the lower limbs, including medial tibial stress syndrome [2–4], patellofemoral pain [5] and ankle/ft injuries due to overuse [6], which arises from tension forces exerted by excessive movement. Kotari et al [7] shows that children with flat feet, for example, are more susceptible to both the emergence of pain and discomfort in the ankle and knee.

Postural changes in the feet can cause pain and discomfort in specific areas of the foot (e.g. forefoot, midfoot, and rearfoot) that, over time, can cause injuries due to changes in the force and pressure on the sole of the foot, resulting in areas of overloads. A lower MLA, or pronated foot, features a medial overload of the foot, which can lead to the transfer of large forces to proximal areas, such as the knee, hip and lumbosacral spine [8]. The increased MLA in the supinated foot leads to larger lateral plantar overloads, which can induce subtalar joint stiffness and therefore place a higher burden on the regions of the forefoot and hindfoot [9, 10].

With regard to changes in plantar support and possible susceptibility to the development of lesions [4] (e.g. medial tibial stress syndrome, patellofemoral pain, bone stress reactions), the foot has been the object of many studies [1, 6–8, 11] covering the period from childhood into adolescence. It has been noted that during the musculoskeletal maturation process, the female foot grows rapidly in the first two years after birth, and then at a uniform rate up to the age of twelve. In contrast, there is a growth surge in male feet in the period between 12 and 15 years of age [12].

Furthermore, with regard to the growth phase, another study has found differences in the morphology of the foot between the sexes at ages seven, nine, eleven, fourteen and fifteen years, in which boys had increased rates of lower MLA’s when compared with girls of the same age group [13]. Most of these changes, and the influence of age and gender, are explained by the increase in body mass index (BMI), an intrinsic factor that apparently results in the lowering of the MLA [14].

One way to check the influence of intrinsic risk factors is through the assessment of foot type through the MLA. To evaluate foot posture, both direct and indirect methodologies are used. The indirect form most used from the clinical perspective is performed by taking a footprint on a paper sheet. However, it is important to note that this form of evaluation considers only the midfoot and does not provide information about the positions of the rearfoot and the forefoot. Another approach to evaluating standing foot posture is offered by the FPI-6 [6, 15–17]. This methodology is valid, reliable, multidimensional, and easily accessible for health professionals in a clinical context. Furthermore, its use does not require sophisticated equipment. In addition, the multidimensionality of this approach allows the evaluation of the hindfoot, the midfoot and the forefoot in all planes of motion [15].

However, despite being a valid and reliable tool, there are as yet no studies that include descriptive and normative FPI-6 data for adolescents. Only one study [18] has investigated this population from age three to seventeen as part of a study of normative values for the adult population. The study noted that adolescents had more pronated feet than adults. However, the authors did not extrapolate the results separately for the different age groups as they were analyzing a particularly wide range of growth. One of the issues highlighted in the literature is the difference in children’s foot posture (from 3 to 9 years of age) and in adolescents (from 10 to 19 years of age) [12, 19]. It is therefore important to analyze the data according to the age group of the subjects.

Identifying the parameters that affect the normal development of the foot during adolescence facilitates the development of an understanding of important risk factors related to any misalignment of the feet, as well as of other dysfunctions (e.g. musculoskeletal disorders, such as sprains or patellofemoral pains) or foot injuries. In addition, an understanding of foot posture in adolescents according to FPI-6 criteria may yield values for reference about the anthropometric characteristics of the feet. Knowledge of these parameters will support more accurate therapy treatments for the rehabilitation of foot anthropometric changes, such as pronation and supination.

Thus, the primary aim of this study was to characterize foot posture in school children aged 10 to 14 years, and the secondary objective was to investigate the influences of age, gender and BMI on foot posture. The main hypothesis of this study is that differences will be observed in the FPI-6 values, depending on age, gender, and BMI, and the highest score of FPI-6 will occur in male adolescents with a high BMI and younger age, because some studies in the literature [13, 14] that used different methodologies have demonstrated that the pronated foot is more prevalent under these conditions.

## Methods

### Location and population study

This is a descriptive cross-sectional study with a convenience sample. Data were collected in public schools, between 2013 and 2014, administered by the School Board of Mogi Mirim—Amparo and Pedreira—in São Paulo, Brazil. The study was approved by the Ethics Committee of the Medical School of the University of São Paulo (protocol number 254/12).

Before conducting the evaluation, examiners traveled to the schools and presented the study to the principals, teachers and students. Next, informed consent forms were distributed to the adolescents, who delivered them to their parents. On another day, the informed consent forms, which had been signed by both the parents and the children, were collected, and the adolescents were evaluated in an isolated room in the school.

Anthropometric data such as height, weight and BMI were collected. For BMI classification the Cole index was used [20, 21].

The inclusion criteria was adolescents were between 10 and 14 years of age with the informed consent signed by parents and participant. Exclusion criteria included the presence of congenital or acquired deformities on the foot and, leg length discrepancy of more than or equal to 1.5 cm and neurological diseases.

### Calculation of the sample size

The sample-size calculation assumed a linear regression. Previously-collected data (researchers’ database) were used to estimate the variance of the response (FPI-6 score) with an estimated value equal to 2.67.

A factorial design based on age, gender and BMI was used as a reference for the information, which aimed to detect a mean difference of 1 in the mean scores of the subpopulations in this study (i.e. gender, age and BMI). The possibilities of two types of error were controlled for: type I (rejecting that the population means these groups are the same) and type II (accepting that the average population of these groups are the same). The type II error probability was defined as power. A number of combinations were three: age, gender and BMI, used to obtain the following sample size: 95% power with a type I error probability of 5% resulting in 1364 subjects.

### Foot posture index (FPI) measurements

A total of 1394 subjects from 10 to 14 years of age participated voluntarily in the study (921 girls and 473 boys), with a total of 2788 ft. The females were more interested in the study, for this reason there was discrepancy between gender groups.

**Fig. 1:**
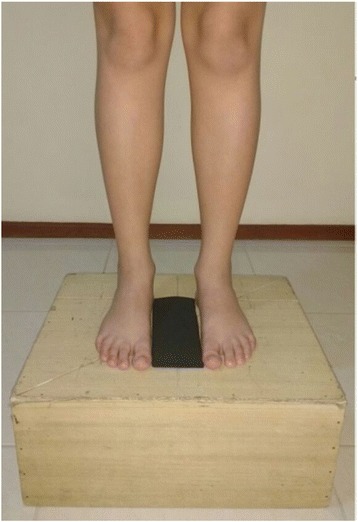
Foot posiotining

Each adolescent was instructed to stand barefoot on a wooden base 19 cm high, 37 cm wide, and 44 cm long. The base had a line 10 cm from its rear edge for positioning the calcaneus, and another line that divided it into two equal parts and which intersected with a third line, forming an angle of 45°. A rectangle of ethyl vinyl acetate (EVA) 7.5 cm x 20 cm was positioned in the middle of the central line to allow standardization of the distance between the feet (Fig. 1).

All subjects were instructed to position the upper limbs along the trunk with eyes directed forward, without any movement such as inclinations and rotations that could interfere with the measurement. FPI-6 criteria were used to quantify foot posture. This form of evaluation has six anatomical criteria as previously outlined by Redmond and colleagues [15], each of which were graded from 0 (neutral), to +1 or +2 (pronated) and −1 or −2 (supinated). The FPI-6 assessment was conducted by two previously trained physical therapists.

Intra- rater reliability was tested in 248 adolescents with a seven-day interval. Inter-rater reliability was tested in 280 subjects on different days, according to the logistics of research.

Scores for each item were added up. A total score of 0 to +5 ranks as normal, +6 to +9 as pronated, 10+ as highly pronated, −1 to −4 as supinated, and −5 to −12 as highly supinated. The final score is a number between −12 and +12 (highly supinated and highly pronated, respectively) [15].

### Statistical analysis

Initially the data were assessed for normality using the Shapiro-Wilk test. Descriptive statistics (mean and standard deviation) were calculated.

To investigate and compare the scores of FPI-6 scores with regards to age and BMI, analysis of variance (ANOVA) was used followed by post hoc Tukey. To compare the FPI-6 items with regard to gender, an independent student *t test* was used. All data were analyzed using SPSS version 21.0 and the 5% significance level [22].

Intra and inter-rater reliability was based on Cohen’s Kappa coefficient and classified as previously described [23].

## Results

Intra-rater reliability was classified as substantial (Kappa = 0.62) and inter-rater reliability was classified as moderate (Kappa = 0.52) [23].

The two cities had essentially the same level of human development; thus, all studied adolescents had similar environmental and socio-economic conditions and came from urban areas. The anthropometric characteristics of the groups (female and male) were similar (Table 1), with a median height of 1.55 ± 0.84 cm for females and 1.56 ± 0.12 cm for males (*p* = 0.234), and a median weight of 50.21 ± 12.53 kg for females and 49.29 ± 13.64 kg for males (*p* = 0.207). However, there was a difference in the BMI of the adolescents, with females showing a higher rate at 20.46 ± 4.03 kg/m^2^ when compared with males at 19.81 ± 3.76 kg/m^2^ (*p* = 0.04).

**Table 1:** Mean, standard deviation and *p*- value of the comparison of anthropometric data in adolescents for sex

Anthropometrics	Female (66%) Mean ± SD	Male (34%) Mean ± SD	*p*-value
Height (m)	1.55 ± 0.84	1.56 ± 0.12	0.234
Weight (kg)	50.21 ± 12.53	49.29 ± 13.64	0.207
BMI (kg/m^2^)	20.46 ± 4.03	19.81 ± 3.76	0.004*

*Independent student t test: significant difference between groups (*p* < 0.05). SD: standard deviation

When comparing FPI-6 scores according to gender (Table 2), it can be observed that the right foot in males has a higher tendency to pronate compared to the female foot (3.09 ± 2.81 versus 2.69 ± 2.61, respectively: *p* = 0.037).

**Table 2:** Mean, standard deviation and *p*-value of the foot posture index (FPI-6) comparison between gender

FPI	Side	Female (66%) Mean ± SD (CI)	Male (34%) Mean ± SD (CI)	*p*-value
Total score	R	2.69 ± 2.61 (4.57; 0.65)	3.09 ± 2.81 (4.77; 0.85)	0.037*
	L	3.47 ± 2.72 (4.68; 0.76)	3.76 ± 2.80 (4.76; 0.84)	0.067

*Independent student t test: significant difference between groups (*p* < 0.05). Side R: right and L: left. SD: standard deviation

With regard to age (Table 3), there were differences between the 11-year and 13-year age groups for the left foot only (*p* = 0.024), with 11-year-old adolescents achieving higher scores (4.16 ± 2.63 versus 3.27 ± 2.79). When compared by age and gender (Table 4), there were no differences between the male and female groups.

**Table 3:** Mean, standard deviation and *p*-value of the FPI-6 comparison in adolescents with different age groups (10 to14 years)

FPI-6	Side	10 years 8% Mean ± SD (CI)	11 years 22% Mean ± SD (CI)	12 years 26% Mean ± SD (CI)	13 years 25% Mean ± SD (CI)	14 years 19% Mean ± SD (CI)	*p*-value
Total score	R	2.74 ± 2.22 (4.18; 0.26)	3.91 ± 2.63 (4,59; 0.67)	3.01 ± 2.77 (4.73; 0.81)	2.58 ± 2.79 (4.75; 0.83)	2.91 ± 2.88 (4.84; 0.92)	0.141
	L	3.65 ± 2.38 (4.34; 0.42)	4.16 ± 2.63 (4.59; 0.67)	3.61 ± 2.78 (4.74; 0.82)	3.27 ± 2.79 (4.75; 0.83)	3.51 ± 2.97 (4.93; 1,01)	0.024^11–13^*

*ANOVAs – post-hoc Tukey: significant difference between groups (*p* < 0.05). Side R: right and L: left. SD: standard deviation

**Table 4:** Mean, standard deviation and *p*-value of the FPI-6 comparison among adolescent females and males with different age groups (10 to 14 years)

Female							
FPI-6	Side	10 years 8% Mean ± SD (CI)	11 years 22% Mean ± SD (CI)	12 years 25% Mean ± SD (CI)	13 years 24% Mean ± SD (CI)	14 years 19% Mean ± SD (CI)	*p*-value
Total score	R	2.55 ± 2.88	4.25 ± 2.54	2.75 ± 2.56	2.39 ± 2.69	2.77 ± 2.78	0.241
		(4.84; 0.92)	(4.50; 0.58)	(4.71;0.60)	(4.65;0.73)	(4.74;0.82)	
	L	3.63 ± 2.56	4.12 ± 2.61	3.49 ± 2.43	3.15 ± 2.69	3.34 ± 2.91	0.107
		(4.52; 0.60)	(4.57; 0.65)	(4.39;0.47)	(4.65;0.73)	(4.87;0.95)	
Male							
FPI-6	Side	10 years 9% Mean ± SD (CI)	11 years 21% Mean ± SD (CI)	12 years 27% Mean ± SD (CI)	13 years 23% Mean ± SD (CI)	14 years 17% Mean ± SD (CI)	*p*-value
Total score	R	3.06 ± 2.12	3.37 ± 2.73	2.93 ± 2.89	2.97 ± 2.99	3.20 ± 3.08	0.793
		(4.08;0.16)	(4.69; 0.77)	(4.85;0.93)	(4.95;1.03)	(5.04;1.12)	
	L	3.67 ± 2.06	2.73 ± 2.73	3.61 ± 2.79	3.55 ± 2.97	3.88 ± 3.08	0.559
		(4.02;0.10)	(4.69; 0.77)	(4.75;0.83)	(4.93;1.01)	(5.04;1.12)	

ANOVAs – post-hoc Tukey: significant difference between groups (*p* < 0.05). Side R: right and L: left. SD: standard deviation

With regards to BMI categories (Table 5), there was a difference in the right foot (*p* = 0.001) in the groups normal and obese. This was also the case for normal and overweight groups (*p* = 0.039) and normal and obese in the left foot (*p* = 0.001).

**Table 5:** Mean, standard deviation and *p*-value of the FPI-6 comparison between the different body mass index (BMI) values (underweight, normal, overweight, and obese) in adolescents

FPI-6	Side	0- Underweight 8% Mean ± SD (CI)	1-Normal 61% Mean ± SD (CI)	2- Overweight 22% Mean ± SD (CI)	3-Obese 7% Mean ± SD (CI)	*p*-value
Total score	R	2,83 ± 2,8 (4.76; 0.84)	2,98 ± 2,6 (4.56; 0.64)	2,69 ± 2,6 (4.56; 0.64)	1,96 ± 2,9 (4.86; 0.94)	0,001^1–3*^
	L	3,43 ± 2,8 (4.76; 0.84)	3,80 ± 2,7 (4.66; 0.74)	3,34 ± 2,6 (4.56; 0.64)	2,60 ± 2,9 (4.86; 0.94)	0,039^1–2*^ 0,001^1–3*^

*ANOVAs – post hoc Tukey: significant difference between groups (*p* < 0.05). Side R: right and L: left. SD: standard deviation

## Discussion

This study presents the descriptive and normative values of FPI-6 criteria in a population of adolescents aged between 10 and 14 years. Overall, the feet in this study were classified as normal, with some degree of pronation, with the boys showing higher scores than girls. Differences were also observed between the 11-year and 13-year age groups, and with BMI. The group classified as having normal weights had higher FPI-6 scores than other BMI groups, indicating a tendency towards pronation. Thus, our hypotheses were only partially confirmed. Although differences were apparent between gender, age, and BMI groups, the highest scores for FPI-6 (a greater tendency toward pronated feet) were not necessarily experienced by younger adolescents or by adolescents with higher body mass.

The total FPI-6 score in the female group averaged 2.69 ± 2.61 for the right foot and 3.47 ± 2.72 for the left foot. In males, the average was 3.09 ± 2.81 and 3.76 ± 2.80 for right and left feet, respectively. Redmond et al. [18], when evaluating subjects from 3 to 17 years of age, obtained results similar to ours with average values of 3.7 ± 2.5 in the evaluation of a single member. Another study [24], developed in children (6–12 years-old) with similar objectives to those of the present study also found that the scores of FPI-6 were slightly higher in boys than in girls, with values of 3.93 ± 2.99 versus 3.61 ± S2.86 for right foot and 4.00 ± 2.96 and 3.74 ± 2.87 for the left foot. However, direct comparison of the results is hindered by the fact that these authors analyzed a different age range. Foot posture is known to differ during the period of growth and maturation [25]; that is, children’s feet differ from those of adolescents. Therefore, the two groups should not be compared. In this context, we emphasize the differential of this study, which evaluated only the period of adolescence.

Differences were observed between the sexes, with boys more likely to have a pronated foot posture. Research in children and adolescents with different methodologies corroborates this finding [11, 13, 26]. A possible explanation is that boys require more time for resorption of the fat pad along the MLA. As a result, males’ feet reach maturity later than girls. This may have contributed to the fact that these adolescents had a postural adjustment mechanism that was more pronated both in static and dynamic form. However, this mechanism may have disappeared by adulthood. In the studies of Redmond et al. [18] and Sanchez-Rodriguez et al. [27] no significant differences in FPI-6 scores were found between the sexes.

Another important point observed in this study was the difference in left foot posture between the 11-year and 13-year age groups. It is known that there is an asymmetry in the human body, such that the left foot is more related to the bearing function, while the right foot is more related to the propulsion of the body during locomotion [28]. In this sense, because postural assessments occur when a subject is in a static posture, the support foot is likely to take on more pronated features due to the increased weight-bearing function. However, this is only a theory, since it cannot be confirmed from our results. Furthermore, it has been noted that foot posture varies from childhood to adolescence, stabilizing at around 15 years [12]. It is likely that this difference is inherent in the development and maturation of the musculoskeletal system. In addition, it is important to emphasize the ability of the FPI-6 criteria to detect postural differences between age groups, as observed previously [18].

The relationship between high BMI and a pronated foot is not in agreement with the literature [14, 29–31]. Evans and Karimi [30] studied the relationship between foot posture and BMI in 728 subjects from 3 to 15 years of age and found no association between increased BMI and a pronated foot. Another study, which used the FPI-6 criteria in 140 volunteers aged seven to ten years of age, found that the heavier children had fewer pronated feet [31]. Adult data supports this finding with no correlation between foot posture and BMI [18, 27]. On the other hand, some authors argue that subjects with a higher BMI present a greater degree of pronated and wider feet [14]. This may affect the calculation of the indices and subsequent classification, particularly in methodologies that take the footprint into account.

In comparing BMI categories, there was a significant difference for the right foot between obese and normal groups. For the left foot, this was true for normal, overweight and obese groups. Interestingly, the subjects of this sample with higher BMI did not show the most pronated foot type and the foot becomes less pronated with increasing BMI, as observed by Gijon-Nogueron in children (6–12 years-old) [32]. A possible reason for this finding is that, due to excess body weight, these adolescents have probably adopted an adaptive postural mechanism to improve and/or maintain balance. It has been shown in the literature that high BMI and poor foot posture are associated with a balance deficit [33–35].

It is necessary to emphasize the importance of understanding the postural changes to the foot during adolescence. At this age, preventive rehabilitation can be carried out, preventing functional impairments in adulthood. About 23% of adults have pronated feet [36]. In addition, it is important to provide a reference standard that can assist in the design of what is or is not to be expected in particular age groups, with regard to gender and BMI, thus facilitating clinical approaches, that is, treat or not treat a pronated/supinated child’s foot, for example.

Our study shows the FPI is a good outcome measure for foot posture in adolescents, because the intra-rater reliability was considered to be substantial and inter-rater reliability was considered to be moderate. Other authors have presented similar results; for example, Morrison et al. [17] found an excellent inter-rater reliability (kw = 0.86) for their study of 30 subjects between 5 and 16 years old, and Evans et al. [37] found a good intra-rater reliability (ICC = 0.93-0.94) and inter-examiner reliability (ICC = 0.79) for their study of 30 healthy subjects between 7 and 15 years old.

In summary, the major contribution of this study is to describe and characterize the foot types using FPI-6 criteria in a population of adolescents aged between 10 and 14 years and to compare this with intrinsic factors such as age, gender and BMI. In addition, an understanding of foot posture in adolescents according to FPI-6 criteria may yield values for reference concerning the anthropometric characteristics of the feet. We hope that the FPI-6 will serve as a basic instrument to detect postural changes, provide more accurate diagnoses, and support possible treatments for adolescents.

A number of limitations must be acknowledged, including discrepancies in the sample sizes with respect to sex and BMI classifications, which made more direct and specific discussions about changes in foot posture challenging. In addition, the study was designed using a convenience sample, making it difficult to extrapolate the data; however, we believe that the data can be extrapolated to populations with similar characteristics: that is, adolescents in urban areas and public schools with similar levels of human development. For future research, we suggest studies verifying the changes that occur in the foot over time during adolescence to determine how the foot posture can change during this period of life and, thus, verify that, at this stage, children are more likely to have musculoskeletal disorders due to their foot posture.

## Conclusion

The feet of most adolescents in this study were classified as normal with only some degree of pronation, particularly for the boys. For the left foot, there were differences between the 11- and 13-year-old age groups, with the 11-year-old group presenting greater tendencies toward pronated feet when comparing posture with age. In addition, adolescents with normal weights had higher FPI-6 scores than adolescents from the other BMI groups (i.e. underweight, overweight and obese). This suggests that increased BMI does not result in a prone foot posture. The data from this study contribute to the current literature by reporting FPI-6 values for the school children population.

## Abbreviations

ANOVA: Analysis of variance
BMI: Body mass index
EVA: Ethyl vinyl acetate
FPI-6: Foot posture index
L: Left
MLA: Medial longitudinal arch
R: Right
SD: Standard deviation
